# Evaluation of multiple protein docking structures using correctly predicted pairwise subunits

**DOI:** 10.1186/1471-2105-13-S2-S6

**Published:** 2012-03-13

**Authors:** Juan Esquivel-Rodríguez, Daisuke Kihara

**Affiliations:** 1 Department of Computer Science, College of Science, Purdue University, West Lafayette, IN 47907, USA; 2 Department of Biological Sciences, College of Science, Purdue University, West Lafayette, IN 47907, USA

## Abstract

**Background:**

Many functionally important proteins in a cell form complexes with multiple chains. Therefore, computational prediction of multiple protein complexes is an important task in bioinformatics. In the development of multiple protein docking methods, it is important to establish a metric for evaluating prediction results in a reasonable and practical fashion. However, since there are only few works done in developing methods for multiple protein docking, there is no study that investigates how accurate structural models of multiple protein complexes should be to allow scientists to gain biological insights.

**Methods:**

We generated a series of predicted models (decoys) of various accuracies by our multiple protein docking pipeline, Multi-LZerD, for three multi-chain complexes with 3, 4, and 6 chains. We analyzed the decoys in terms of the number of correctly predicted pair conformations in the decoys.

**Results and conclusion:**

We found that pairs of chains with the correct mutual orientation exist even in the decoys with a large overall root mean square deviation (RMSD) to the native. Therefore, in addition to a global structure similarity measure, such as the global RMSD, the quality of models for multiple chain complexes can be better evaluated by using the local measurement, the number of chain pairs with correct mutual orientation. We termed the fraction of correctly predicted pairs (RMSD at the interface of less than 4.0Å) as *fpair *and propose to use it for evaluation of the accuracy of multiple protein docking.

## Introduction

An essential part of protein structure prediction is to establish methods to evaluate computed models. For single protein structure prediction, the research community, which is partly driven by the Critical Assessment of Protein Structure Prediction (CASP), often uses the global RMSD as well as its variant, the GDT-TS score [1]. On the other hand, the protein docking community, which is partly led by the Critical Assessment of Prediction of Interactions (CAPRI) [2], often uses the RMSD at the docking interface named the iRMSD and the fnat (fraction of correctly predicted native contacts). The iRMSD and the fnat are originally designed to evaluate the accuracy of pairwise protein docking models.

Earlier works on multiple protein docking used the global RMSD for evaluating the model accuracy [3-7]. Of course the global RMSD, the iRMSD, or the fnat can be used to identify accurate models of multiple chain complexes. However, since the sizes of the whole multiple chain complexes can be much larger than single protein structures or pairwise protein complexes, the usefulness of multiple chain complex models can be better understood and evaluated if the global quality measures are complemented by additional measures that quantify local accuracy of models.

Here, we generated decoys of multiple protein complexes using Multi-LZerD [8,9], a multiple protein docking method developed in our group. We analyzed the decoys in terms of the number of pairwise interactions in whole multiple chain complexes that have been accurately predicted, that is, pairs with an iRMSD of less than 4Å (the iRMSD only takes into account atomic coordinates at the docking interface region). We show that, even when the apparent overall RMSD of a multi-chain complex seems to be high, in many cases there are several accurately predicted pairwise interactions. Such models would be still useful for certain purposes since they contain a significant number of docking interface residues that are correctly placed relative to their interacting chains. We highlight this by proposing a new accuracy measure for multiple docking, named *fpair *(fraction of pairwise hits) that accounts for the proportion of correct pairwise predictions among all chain pairs in a whole multiple chain complex.

## Methods

We used Multi-LZerD [8,9] to construct decoys of various global accuracy (RMSD) ranges for three multiple protein complexes: 1A0R, 1NNU, and 1I3O, which are 3, 4, and 6 chain complexes. Here we briefly explain the Multi-LZerD algorithm. Multi-LZerD takes the 3D structure of component chains of a multiple chain complex as input, and first employs the LZerD algorithm [10], a pairwise protein docking method developed in our group, to generate a few tens of thousands pairwise docking conformations for each chain pair. A characteristic of LZerD is that it uses the 3D Zernike descriptors [11,12], a series expansion of a 3D function, to represent protein surface shape and to identify shape complementarity of surfaces.

A conformation of a multiple chain complex can be uniquely specified by denoting which pairwise docking decoys to combine from the pool of the pre-computed pairwise decoys by LZerD. Multi-LZerD explores different conformations of the whole complex by altering pairwise decoys using a genetic algorithm [13]. The fitness function used to evaluate decoys is a linear combination of physics-based scoring terms. After 3000 generations, Multi-LZerD finally outputs 200 models of the complex. Clustering is applied at the end of every generation [14], thus, the number of final set of decoys is less than 200.

## Results

### Correct pairwise interactions in decoys

Three protein complexes of different number of chains, 3 (PDB ID: 1A0R), 4 (1NNU), 6 (1I3O) were used in this study. These decoys are classified by the RMSD into 6 classes, 0-4Å, 4-8Å, 8-12Å, 12-16Å, 16-20Å, and 20Å or larger. Additionally, for each decoy, we computed the iRMSD for all pairs of chains included in the complex by comparing each pair in the decoy to the corresponding pair in the native structure. If the iRMSD is lower than 4Å the pair is considered a hit. An iRMSD of 4Å is a criterion of acceptable prediction for pairwise docking used in the CAPRI. Using the pairwise hit count in a whole complex decoy we calculate the *fpair *value, which is defined as the fraction of pairwise combinations that are considered hits, from the total pairwise combinations:

$$fpair=\frac{\underset{p\in P}{\sum }I\left(iRMSD\left(p\right)<4Angstrom\right)}{\left(\begin{array}{c} N\\ 2 \end{array}\right)},$$

where *P *is the set of all pairwise combinations. *I *is the indicator function that represents 1 if the predicate is true (i.e. iRMSD is smaller than 4Å) and 0 otherwise.

For example, a 3-chain complex with chains A, B, and C has 3 pairwise combinations, A-B, A-C, and B-C. A-B from a predicted structure is superimposed onto A-B structure taken from the native complex structure, without taking the chain C into account. The same process is repeated for A-C and B-C. If 2 out of these 3 pairwise combinations are hits then *fpair *is 2/3 = 0.67 for the predicted complex structure.

**Figure 1:**
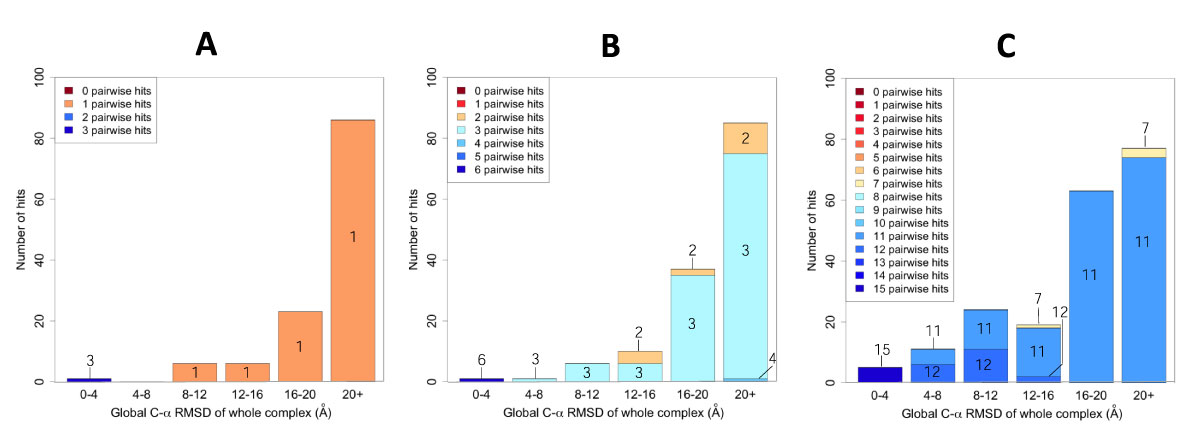
**Count of pairwise hits in multiple protein docking predictions**. Generated decoys of 3 protein complexes are classified based on the global RMSD to the native structure and the number of pair conformations with iRMSD of less than 4Å was counted. **A**, 1A0R (3 chains); **B **1NNU (4 chains); **C**, 1I3O (6 chains).

Figure 1 shows the distribution of the global RMSD of the decoys classified into the 6 classes and the number of the pairwise hits included in the decoys. Figure 1A shows the results for 1A0R, a 3 chain complex. There was one decoy in the range of 0-4Å (leftmost bar). For this decoy, all 3 chain pairs have iRMSD < 4Å. No decoys are found in the 4-8 Å global RMSD range. Interestingly, all the decoys in the range of 8-12Å (6 decoys), 12-16Å (6 decoys), 16-20Å (23 decoys) and 20Å+ range (86 decoys) have one pairwise hit, which gives an fpair of 0.33. Thus, even when the global RMSD is very large, e.g. 20+Å, 2 out of 3 chains are correctly predicted and the location of additional one chain made the apparent global RMSD large.

Figure 1B shows the results for 1NNU, a 4 chain complex (thus there are 6 = 4 × 3/2 chain pairs). Out of 10 decoys in the global RMSD range of 12-16Å, 4 decoys contain 2 pairwise hits (fpair of 0.33) while the other 6 decoys contain 3 pairwise hits (fpair of 0.5). Even in higher global RMSD ranges, there are still chain pairs that are correctly predicted. In the range of 16-20Å there are 2 decoys with 2 pairwise hits and 35 with 3 hits. Finally, in the 20Å+ range 10 decoys have 2 hits, 74 decoys have 3 hits and 1 decoy has 4 pairwise hits (fpair 0.67).

Figure 1C presents the results for 1I3O, a 6 chain complex. In the range of 0-4Å, all 5 decoys have the maximum number of pairwise hits, 15, i.e. a fpair value of 1.0. In the next three classes, 4-8Å, 8-12Å, there are significant number of decoys with 12 and 11 hits, which yield fpair of 0.8 and 0.73, respectively. At the range of 12-16Å, there is one decoy with 7 hits (fpair of 0.47). Finally, it is notable that the last 2 classes, 16-20Å and 20+Å, are dominated by decoys with 11 hits, except for 3 decoys in the 20Å+ range that have 7 hits.

### Examples of decoy structures

**Figure 2:**
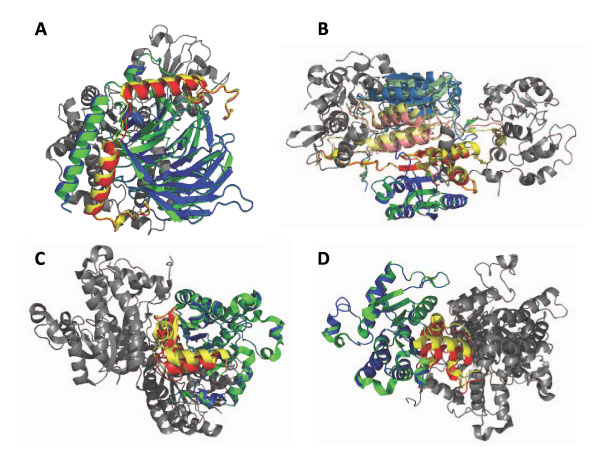
**Examples of accurately predicted chain pairs in globally inaccurate decoys**. **A**, a decoy for 1A0R (3 chains) with a global RMSD of 15.12Å but shows an RMSD of 0.51Å for the B-G sub-complex. **B**, a 1I3O decoy (6 chains) with a global RMSD of 14.17 Å but yields a correct prediction of chains A, B, C, and D (RMSD: 2.22Å). **C **and **D**, a 15.68Å global RMSD decoy for 1NNU (4 chains), show the alignment of 2 sub-complexes correctly predicted on their own (A-C and B-D are superimposed in the panel **C **and **D**, respectively).

In Figure 2 we show examples of decoys with a high global RMSD but contain correctly predicted chain pair conformations. Figure 2A shows a decoy for 1A0R, of which global RMSD is 15.12Å. Despite of the seemingly large RMSD, relative positions of two chains B (green/blue for native/predicted position of the chain) and G (yellow/red for native/predicted) are very well predicted with an iRMSD of 0.51Å. A similar case can be seen in Figure 2B, where a decoy for 1I3O is presented. The global RMSD of this decoy is 14.17Å; however, relative positions of 4 chains out of 6 chains, chains A (green/blue for native/predicted), B (yellow/red), C (pale green/pale blue), and D (pale yellow/pale red) are very well predicted with a RMSD of 2.22Å. Finally, Figures 2C and 2D show a decoy for 1NNU (15.68Å global RMSD). The decoy is shown from two different angles to clearly show the pairs of chains A-C and B-D, both of which are predicted within iRMSD of 4.0Å.

## Conclusions

We have shown that, while the global C-α RMSD is a clear indication of high quality predictions for multiple protein docking, a predicted structure with a higher RMSD should not be simply discarded as unsuccessful prediction since in many cases such decoys contain correctly predicted subcomplexes. We propose a measure named *fpair* for assessing the fraction of correctly predicted pairs among all pairs in a whole complex. By using *fpair *one can distinguish models that have partially accurate subcomplexes from models with the same global RMSD but do not contain any correctly predicted pairs. *fpair *will complement the traditional global measurements like RMSD and fnat for evaluating quality of models for multiple protein complexes.

## Competing interests

The authors declare that they have no competing interests.

## Authors' contributions

JER developed the multiple protein docking prediction method, Multi-LZerD, performed the computational experiments and wrote the manuscript draft. DK conceived the study and participated in its design and coordination, as well as drafting and finalizing the manuscript. All authors read and approved the final manuscript.
